# Toward the Chemoenzymatic Synthesis of DNA-Encoded Libraries

**DOI:** 10.1021/acscentsci.5c01516

**Published:** 2026-01-06

**Authors:** Daniela Schaub, Alice Lessing, Gerlis von Haugwitz, Fabian Meyer, Jörg Scheuermann, Rebecca Buller

**Affiliations:** † Competence Center for Biocatalysis, 111833 Zurich University of Applied Sciences, 8820 Wädenswil, Switzerland; ‡ Center for Functional Protein Assemblies & Department of Bioscience, TUM School of Natural Sciences, Technical University of Munich (TUM), 85748 Garching, Germany; § Department of Chemistry and Applied Biosciences, 27219 ETH Zurich, 8093 Zurich, Switzerland; ∥ Department of Chemistry, Biochemistry and Pharmaceutical Sciences, University of Bern, 3012 Bern, Switzerland

## Abstract

DNA-encoded libraries (DELs) have become a powerful platform in drug discovery, practiced both by the pharmaceutical industry and academia. Each small molecule contained in a DEL is covalently linked to a DNA tag which serves as an amplifiable barcode facilitating binder identification. However, the chemical diversity accessible in DELs remains limited by the need to perform reactions under conditions that preserve the integrity of the DNA tag. Additionally, chemical reactions must proceed with high efficiency and selectivity to minimize side products and unreacted starting materials, which cannot be removed and may hamper hit identification. Consequently, expanding the DEL chemical space requires the development of methods that combine high reaction performance with DNA compatibility. In this outlook, we highlight the potential of enzymatic catalysis for on-DNA synthesis, which offers a promising route to expand DEL-accessible chemical space.

## Graphical abstract

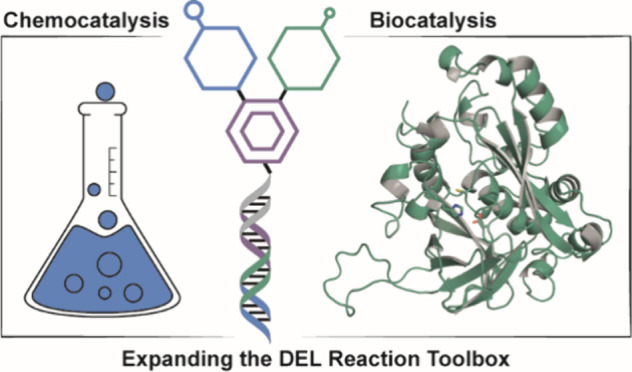

## Introduction

To meet the growing demand for new and improved therapeutics, the pharmaceutical industry dedicates substantial resources to research and development.^1,2^ Yet, identifying small molecules that bind tightly and selectively to pharmaceutically relevant targets remains a major challenge. As part of the drug discovery process, compound libraries are systematically screened to identify small molecules with biological activity and potentially therapeutic properties. Toward this goal, high-throughput screening (HTS) procedures have been developed, enabling the rapid screening of millions of compounds.^3^ However, HTS relies on spatially separated compounds, large material inputs, and the development of target-specific bioassays,^4^ rendering it an expensive approach. Thus, technologies that streamline ligand discovery are of considerable interest, as they have the potential to significantly accelerate the drug development process and reduce cost. For example, the generation of smaller, diversity-oriented compound libraries^5^ and computer-assisted methods^6^ can help to reduce needed resources. Similarly, DNA-encoded chemical libraries (DELs)^7–10^ have been developed as an alternative lead compound identification strategy, representing a powerful and inexpensive approach in drug discovery.

Typical DELs are collections of millions to billions of small molecules, each tagged by a distinct DNA barcode, which enables unambiguous compound identification and allows for the simultaneous screening of large compound libraries at once. Today, DEL technology has yielded ligands for a range of pharmaceutically relevant targets, with several compounds progressing into clinical development,^11^ such as a soluble epoxide hydrolase (sEH) inhibitor,^12^ receptor interacting protein 1 kinase inhibitors,^13,14^ and an autotaxin inhibitor.^15^

## DNA-Encoded Libraries: Synthesis and Selection

DNA-encoded libraries are commonly produced by combinatorial split-and-pool synthesis, which allows the rapid and inexpensive synthesis of very large libraries.^9,10,16–18^ Typically, library synthesis begins with a short, common oligonucleotide connected to a functional handle (often a primary amine) through a linker (collectively called the “headpiece”). A stock solution with the DNA headpiece is distributed into several reaction vessels before a first set of chemical building blocks is individually added and reacted with the functional handle attached to the common oligo. To barcode the chemical moiety anchored to the DNA strand, the oligonucleotide is extended by enzymatic ligation to a specific DNA fragment. Once encoded, the reaction products are pooled again, and further cycles of chemical synthesis and DNA ligation can take place (Figure 1).^19^ In this way, DELs consisting of up to billions of individual compounds can be created, with the DNA tags uniquely identifying each chemical compound in the library.^18,20,21^ For example, a library of approximately three million compounds can be created by reacting 142 × 142 × 147 building blocks in three consecutive DEL synthesis cycles.^22^

While there is no theoretical limitation to the number of synthesis rounds, decreasing library quality stemming from varying chemical yields and the concomitant accumulation of truncated products limits standard DEL synthesis to two to four cycles. Innovative approaches like the synthesis of DELs on solid phase with a final purifying step leading to the selective release of correctly synthesized products have been proposed for constructing high-quality DELs over more cycles.^23^ Furthermore, libraries can be assembled through hybridization of sublibraries, which are synthesized separately on partly complementary DNA strands (dual-display DELs). With this approach, smaller sublibraries can quickly transform into extensive DNA-encoded repertoires, encompassing the desired millions of library members, while maintaining high quality.^24–29^

Once synthesized, the DNA-encoded libraries are used in an affinity-based selection process to identify small molecules that possess a high binding affinity to a biological target of interest (Figure 1).^13–15,30^ During panning, nonbinders are eliminated using washing steps, while binders are retained. Because DNA barcodes can be readily amplified using the polymerase chain reaction (PCR), the selection process relies on only a small number of copies of each compound (∼10^5^)^31^ and hence, DEL synthesis can be conducted at nanomolar scale, reducing material input.^8^ Following the amplification, the barcodes are sequenced using next-generation DNA sequencing leading to the identification of individual binder structures through the analysis of relative abundance.

**Figure 1:**
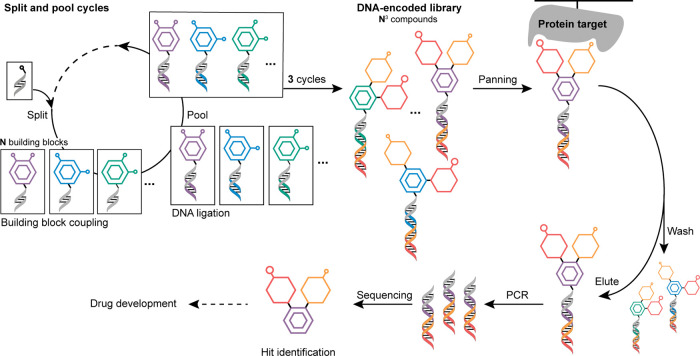
DNA-encoded small molecule library synthesis and screening. In the first step of constructing a DNA-encoded library, the initial common oligonucleotide (headpiece) is split before being coupled to distinct chemical building blocks. Next, the identity of each chemical building block is encoded via a unique DNA fragment, which is added to the DNA headpiece by enzymatic ligation. The reaction products are pooled, and another iteration of splitting, chemical synthesis, and encoding takes place to yield the final DNA-encoded library, in typically two to four overall cycles. The DNA-encoded chemical library is applied in affinity-based selections against an immobilized protein target (panning). Nonbinders are washed off, while binders are subsequently eluted. DNA amplification and sequencing is conducted to identify the binding molecules (hits).

Regardless of the strategy chosen for DEL assembly, the chemistry that can be applied to prepare the library is restricted by the stability of the DNA tag.

Regardless of the strategy chosen for DEL assembly, the chemistry that can be applied to prepare the library is restricted by the stability of the DNA tag. Only a subset of available chemical reactions is compatible with the presence of DNA, limiting the accessible chemical space and library diversity.^32,33^ Besides the need for aqueous conditions to solubilize the DNA tag, the employed reactions must be DNA-orthogonal and avoid or at least minimize DNA damage, such as depurination, cleavage, or base transversions.^33,34^ Split and pool-based combinatorial synthesis further requires that reactions must proceed with high conversions for a wide range of substrates and predominantly lead to the desired reaction product, as the purification of individual conjugates is only feasible before the first pooling step. Incomplete reactions lead to the accumulation of truncated structures within the library. Both side products and truncated structures stemming from low chemical yields can considerably complicate hit discovery during affinity-based selections by introducing noise and leading to false negatives.^23,35^ Considering these synthetic restrictions, biocatalysis could be a particularly attractive addition to the DEL reaction portfolio, as enzymes are powerful catalysts naturally operating under “DNA-friendly” conditions.^32,36^

Considering these synthetic restrictions, biocatalysis could be a particularly attractive addition to the DEL reaction portfolio, as enzymes are powerful catalysts naturally operating under “DNA-friendly” conditions.

With this outlook, we seek to bring attention to this underexplored topic by discussing strategies for chemoenzymatic library synthesis and outline opportunities and challenges that in our opinion will shape future enzyme-based DEL methodologies.

## Biocatalysis for the Synthesis of DELs: Strategies

The first example of enzymatic on-DNA chemistry was reported by Thomas *et al.*, who used glycosyltransferases and oxidases to construct a small carbohydrate library (Scheme 1).^37^ Such a glycoside-based library is an exciting showcase for biocatalysis, as sugar molecules are complex and require highly chemo- and regioselective reaction steps. In this study, chemical ligation was used to attach the initial sugar building blocks onto a double-stranded DNA headpiece (**HP**) before glycosyltransferases were employed to elongate the on-DNA carbohydrate by sialylation or galactosylation. For example, bovine β-1,4-galactosyltransferase (β1,4-GalT) was used together with its donor substrate uridine diphosphate galactose (UDP-Gal) for the galactosylation of DNA-conjugated *N*-acetylglucosamine **1**, leading to *N*-acetyllactosamine **2** (Scheme 1). To create additional diversity, a galactose oxidase (GOase) variant was used to catalyze the regioselective oxidation of the sugar’s primary hydroxyl group to the corresponding aldehydes, leading to 14 enzymatically generated DNA-conjugates. This enzymatic oxidation step enabled the chemical ligation of additional building blocks by hydrazone ligation or reductive amination. While not all enzymatic transformations yielded high conversions (15 – 94%), mass analysis showed that no undesired side or DNA-degradation products were formed, indicating the specificity and DNA compatibility of the biocatalytic reactions.

**Scheme 1:**
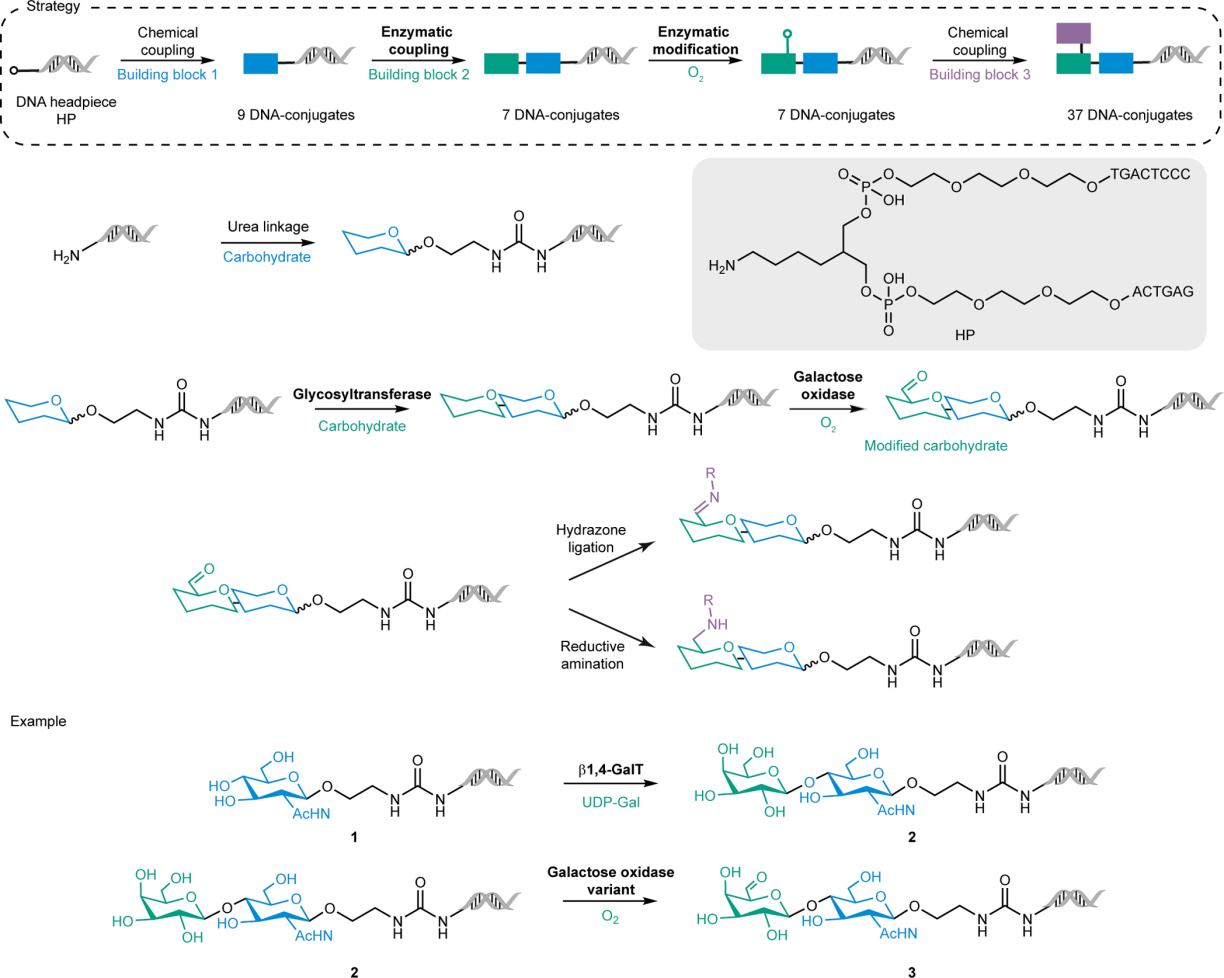
Chemoenzymatic Synthesis of DNA-Conjugated Carbohydrates

In another example, threonine aldolases (TAs) were used to generate DNA-conjugated β-hydroxy-α-amino acids from glycine and DNA-conjugated aldehydes (Scheme 2a).^38^ In analogy to the construction of the chemoenzymatic carbohydrate library, the first building blocks (aldehydes) were chemically attached to the double-stranded DNA headpiece (**HP**) before addition of the second building block glycine via an enzymatic aldol reaction. The enzymatically generated β-hydroxy-α-amino acids (aldol products) served as versatile intermediates for subsequent chemical modifications: Treatment of the DNA-conjugated β-hydroxy-phenylalanine **6** with p-nitrophenylchloroformate (PNPCl) led to the cyclic oxazolidinone product **7** (96% conversion). Other chemical modifications of the enzymatically formed product included amide bond formation (**8** and **9**), or reductive amination (**10**). The threonine aldolases employed in this study only accepted glycine as the nucleophile. To account for this, diversity was created by varying the DNA-linked aldehyde structure by including aromatic, heterocyclic, bicyclic, and aliphatic compounds, resulting in 13 enzymatically generated DNA-conjugates. The stereoselectivity of the applied enzymes was investigated by derivatization of the aldol products with OPA/*N*-Boc-L-cysteine allowing the separation of diastereomers by liquid-phase chromatography. The analysis revealed excellent α-carbon stereoselectivity of the applied enzymes and, to a lower degree, β-carbon selectivity. To study the impact of the linker connecting the small molecule to the DNA headpiece (**HP**), two additional linkers (PEG_4_ and a hydrophobic linker of the same length) were installed and tested. Both linkers negatively affected β-carbon selectivity, most likely due to the additional flexibility introduced. Interestingly, the PEG_4_ linker led to higher yields (89%) compared to its more hydrophobic counterpart (66%) when using a low-specificity l-threonine aldolase (UniProt: P75823) from *Escherichia coli* (*E. coli*).

**Scheme 2:**
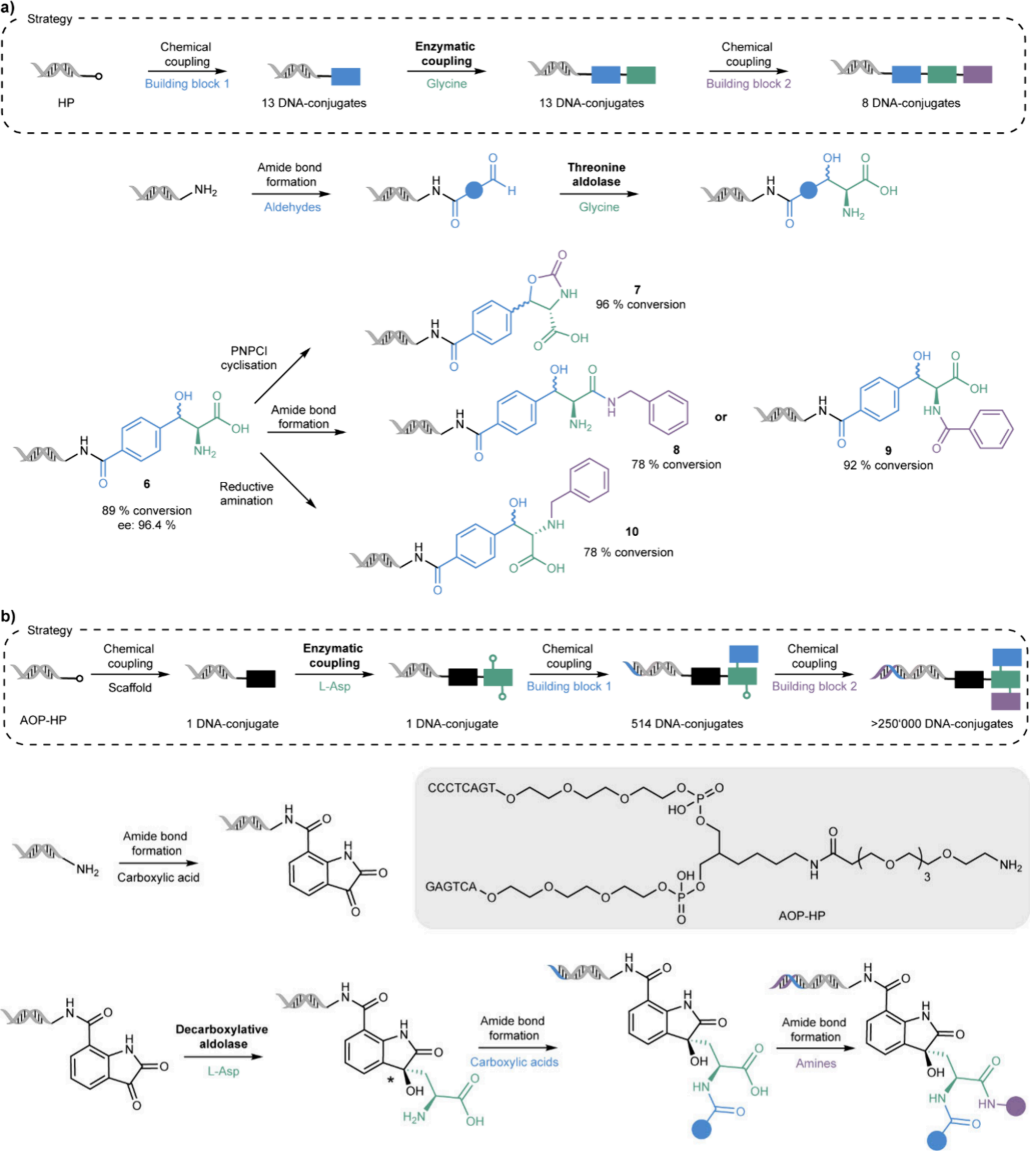
Use of Aldolases for the Chemoenzymatic Synthesis of (a) DNA-Conjugates and (b) a DNA-Encoded Library, with *Predicted Stereoselectivity Based on Reaction Data with Small Molecule Analogues (off-DNA)

Further advancing the application of aldolases in DEL technology, a recent study used the decarboxylative aldolase (ApUstD) from *Aspergillus pseudonomius* to couple a DNA-conjugated indole with the side chain of aspartic acid generating a multifunctional on-DNA scaffold (Scheme 2b).^39^ Due to the narrow substrate scope of ApUstD, its use was limited to initial scaffold generation rather than building block coupling. However, the generated intermediate was further chemically diversified through two additional split and pool synthesis cycles, comprising subsequent amide bond formations with 514 carboxylic acids (cycle 1) and 547 amines (cycle 2) leading to a DNA-encoded chemical library comprising more than 250,000 compounds. The chemoenzymatically constructed library was successfully screened against phosphoglycerate dehydrogenase (PHGDH), a key enzyme in the serine biosynthesis pathway linked to cancer and Alzheimer’s disease, yielding several inhibitors with micromolar IC50 levels (Scheme 2b).

Complementing the above-enumerated investigations, a recent study from our laboratory harnessed a cascade consisting of wildtype and engineered enzymes for on-DNA amide-bond formation.^40^ Combining CoA ligases (CLs) and engineered *N*-acyltransferases (NATs), a diverse set of DNA-conjugated amines was enzymatically coupled with carboxylic acid building blocks (Scheme 3). To build a DNA-encoded library with the enzymatic cascade, an azide-protected building block was first chemically ligated to a single-stranded DNA tag and chemically functionalized with a set of carboxylic acids, including bulky compounds such as oxaprozin and adamantylacetic acid (cycle 1). Subsequent azide deprotection generated the free amine, which was enzymatically coupled to a set of secondary building blocks generating 72 distinct DNA-conjugates (cycle 2) with excellent conversions (on average >91%). Notably, the system was characterized by its flexibility in the pairing of CLs and NATs, in this way enabling access to a broad range of amides in function of the enzymes used. Furthermore, only a modest excess of the carboxylic acid building blocks (5 equiv) was needed to obtain excellent yields (up to 98%) comparing favorably to many studies on chemical amide bond formation on DNA often using an excess of >100 equiv.^18,22,41^ The DNA compatibility of the enzymatic transformation was analyzed by quantitative PCR (amplifiability of DNA barcode postsynthesis) and LCMS, showing no signs of DNA damage occurring or the formation of unwanted side-products.

**Scheme 3:**
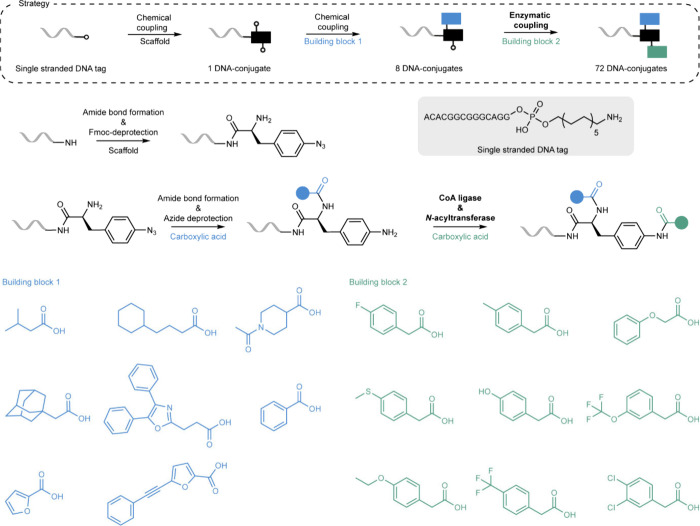
Chemoenzymatic Synthesis of DNA-Conjugates by Amide Bond Formation

Overall, these first studies demonstrate the compatibility of biocatalysis with on-DNA chemistry and highlight key advantages of applying enzymatic transformations on DNA, including regioselectivity (e.g., site-specific oxidation of carbohydrates^37^), chemoselectivity (e.g., selective aldol reaction at an aspartate side chain^39^), stereoselectivity (e.g., α-carbon control in aldol products^38^) and high conversion without the generation of side products or DNA-damage (e.g., high yielding reactions to desired amides^40^). In addition, the examples showcase distinct strategies for integrating enzymatic steps into the DEL synthesis workflow, whether for scaffold formation (aldolase^39^), building block coupling in early but also later cycles of synthesis, for which library purification is not possible anymore (glycosyltransferase, CoA ligase/*N*-acyltransferase), or functional group transformation (oxidation to aldehydes with galactose oxidase).

## Biocatalysis for DELs: Opportunities and Challenges

To be applicable for DEL synthesis, biocatalysts must operate under DNA-compatible reaction conditions, should possess a broad substrate scope, have a controlled chemo-, regio- and enantioselectivity and lead to high conversions. While some of these criteria are intrinsically met by many enzymes, challenges for application may arise from some enzyme’s limited substrate scope and the need to accommodate the bulky DNA tag. In the following paragraphs we comment on these requirements and explore opportunities and challenges when applying biocatalysis to DEL synthesis.

DNA compatibility is a critical factor when selecting reactions for DNA-encoded library (DEL) synthesis. Although it is generally accepted that on-DNA reactions inevitably cause some degradation of the DNA tag, the extent of this damage can vary greatly depending on the reaction conditions. For instance, acylation of a secondary on-DNA amine has been reported to reduce the amount of amplifiable DNA to 17%, whereas exposure to Pd­(PPh_3_)_4_ during O-allyl deprotection resulted in 40% remaining DNA and dropping to as low as 0.1% when the order of reagent addition was altered.^34^ Clearly, the acceptable level of DNA damage depends on several factors, including the amount of starting material and the number of reaction steps in the library synthesis; however, minimizing DNA degradation is generally essential to ensure successful binder identification.

DNA-compatibility of a reaction can be evaluated by quantifying DNA damage occurring during the reaction using quantitative PCR (qPCR), liquid chromatography coupled to mass spectrometry (LCMS), gel electrophoresis, or sequencing. In the context of DEL synthesis and selection, the term DNA damage encompasses all modifications that would disable efficient amplification, prevent DNA elongation, or lead to the loss of stored information.^34^ For example, acidic reaction conditions are known to promote depurination,^42^ while metal catalysts can generate reactive oxygen species, promoting DNA cleavage.^43^ It was even shown that DNA exposure to copper catalysts not only leads to DNA cleavage but also promotes base transversion.^33^ While these types of point mutations might not influence amplification or elongation of the DNA tag, they complicate correct compound identification. The extent of DNA degradation caused by metal salts can vary depending on the metal type, DNA architecture (single- vs double-stranded), and reaction conditions, including temperature and exposure time.^44,45^ These challenges render the use of metal-catalyzed reactions in DEL synthesis difficult and underscore the need for careful reaction condition optimization, or the selection of alternative synthetic strategies to preserve DNA integrity, such as biocatalysis.

Most enzymes have evolved to operate in aqueous environments under mild conditions, such as ambient pressure, temperature, and pH.^46,47^ These operational parameters fit well with the restrictions imposed by the presence of the DNA tag in DEL synthesis. To date, enzymatic transformations using DNA-conjugated substrates have not been associated with DNA degradation.^37,38,40,48^ In our study, for example, we examined DNA compatibility of the enzymatic amide-bond formation by measuring ligation yields (gel electrophoresis) and the amount of amplifiable DNA remaining (qPCR), relative to appropriate controls.^40^ Yet, as some enzymes operate via radical intermediates or require metal cofactors, we suggest that the DNA-compatibility of such biocatalysts must be evaluated on a case-by-case basis.

Substrate scope is potentially one of the major challenges when considering biocatalysis for DEL synthesis.^36,49^ Even with the aim to build a modest DNA-encoded library containing only a few thousand compounds, the applied enzymes must be able to catalyze reactions between hundreds of different molecules.^20^ Encouragingly, analysis of the *E. coli* metabolism showed that 37% of its enzymes exhibit substrate promiscuity and are responsible for catalyzing 65% of *E. coli’s* known metabolic reactions.^50^ Substrate profiling underlines this natural promiscuity: In a comprehensive study, 217 members of the haloalkanoate dehalogenase superfamily were screened against 167 phosphate compounds. The analysis revealed that 50 of the tested enzymes catalyzed phosphate hydrolysis across more than 41 different substrates, with the most promiscuous enzyme able to hydrolyze an impressive 143 substrates.^51^ Reflecting these findings for a bond-forming enzyme, the substrate scope of the amide-bond synthase McbA was investigated by performing 1,100 unique reactions between distinct carboxylic acids and amine substrates, which resulted in product formation in 461 of these reactions.^52^ These data indicate that substrate promiscuity is not an exception but rather an inherent feature of many wildtype enzymes consistent with the notion that natural selection primarily removes activities detrimental to organismal fitness, permitting enzymes to maintain a residual breadth of catalytic function.^53^ Nevertheless, sourcing such enzymes from nature remains a challenge, as there are no general indications for promiscuity based on protein sequence or structure.^54^ For some enzymes, it was shown that substrate promiscuity is linked to flexibility and hydrophobicity of the active site.^55,56^ It is reasonable to assume that larger, more flexible active sites can accommodate a wider range of substrate sizes and shapes, and that hydrophobic active sites promote binding through nonspecific interactions, such as hydrophobic effects, rather than requiring precise electrostatic or hydrogen bonding.^57^ Larger binding pockets may also accommodate bulky DNA-conjugated substrates. Thus, profiling enzymes along the above enumerated criteria might serve as a first guideline to identify promiscuous enzymes to be used in DEL construction.

In addition, while a molecular understanding of promiscuity would be needed for the rational design of “allrounder” enzymes,^58^ it is not a prerequisite for enlarging enzyme substrate scope via directed evolution. Directed evolution is a powerful strategy inspired by natural selection that does not rely on prior structural or mechanistic knowledge.^59^ It involves generating libraries of enzyme variants through mutagenesis, followed by screening to identify variants with improved or altered activity. The most promising variants are then subjected to additional rounds of mutation and selection, gradually enhancing the enzyme’s performance toward the targeted function (Figure 2). Traditionally, this approach is used to improve enzyme function (e.g., activity, solvent or thermal stability), but it also lends itself to evolve variants with broader substrate acceptance when the appropriate selection pressure is applied.^60^ This can be achieved either iteratively, in which case variant libraries are screened in succession for different substrate activities, or in one-pot experiments by applying substrate cocktails.^60,61^ Substrate multiplexing was, for example, employed to engineer a tryptophan decarboxylase (TDC) to act on various tryptophan analogues.^60^ Six substrates were screened in competition against nine site-saturation libraries of the decarboxylases from *Ruminococcus gnavus* (*Rgn*TDC) and the relative as well as total product formation of the variants was determined by LCMS analysis. These screens identified amino acid substitutions with high impact on promiscuity while retaining significant catalytic activity. For several enzyme–substrate pairs, activity increased by more than 50-fold. More recently, a similar approach was undertaken to broaden the substrate scope of a decarboxylative aldolase from *Aspergillus flavus* to generate tertiary γ-hydroxy amino acids from *L*-aspartic acid and a range of carbonyls.^61^ By applying a multisubstrate screening approach to enzyme variant libraries, mutations at positions distant from the active site were identified that had a direct influence on substrate acceptance, underscoring the complexity of substrate promiscuity.^62^ In the context of enzymatic DEL construction, the potential to broaden the substrate scope of decarboxylative aldolases is particularly compelling, as these enzymes have already been utilized for DNA-encoded scaffold assembly (Scheme 2b), where narrow substrate scope was reported as a limitation of the biocatalytic step.^39^

**Figure 2:**
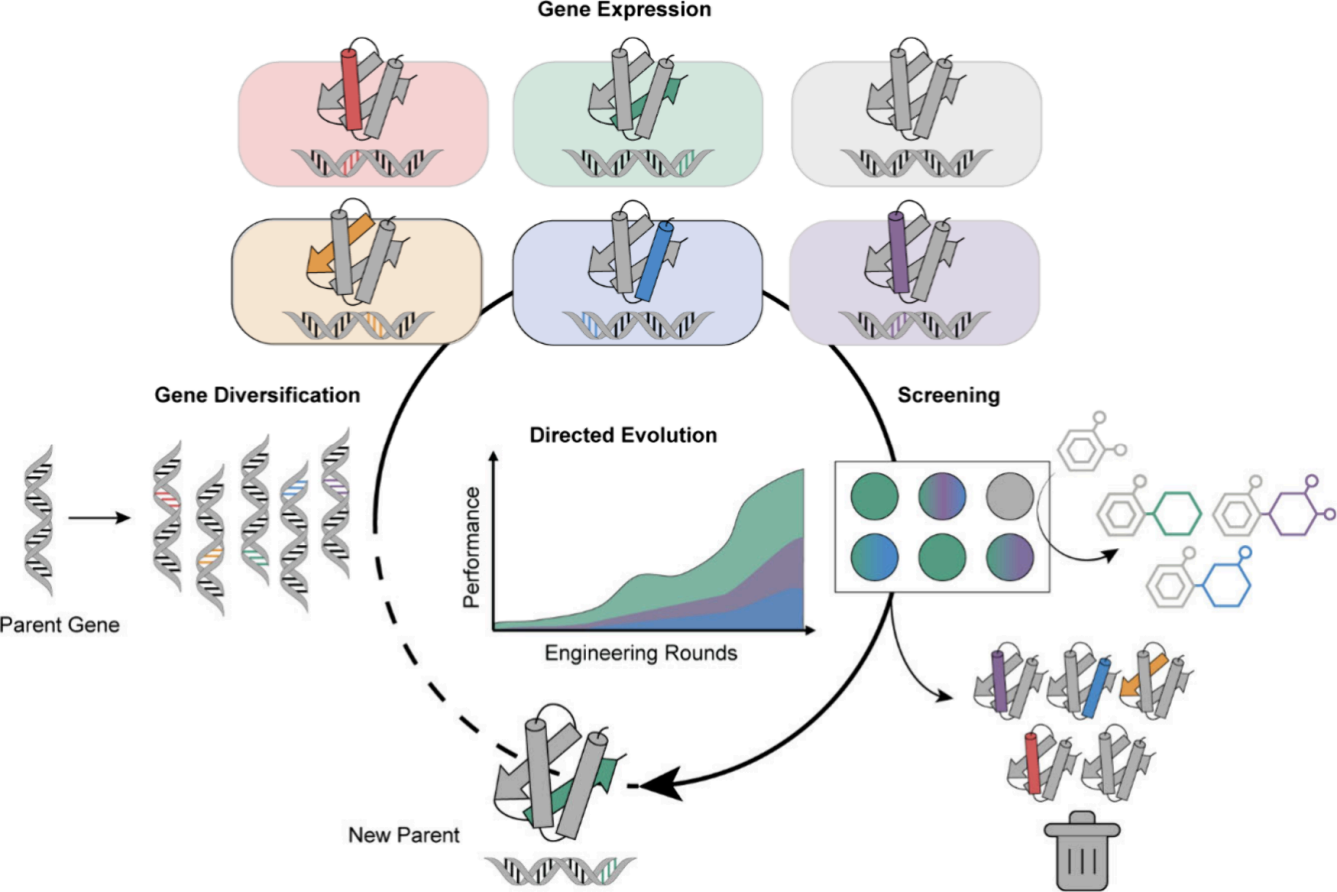
Directed evolution of enzymes. Schematic representation of the directed evolution process, including iterative rounds of mutagenesis and screening. Enzyme variants are generated through random or targeted mutations, followed by screening or selection for improved catalytic properties (depicted target function: substrate promiscuity).

Transitioning from conventional single- to multisubstrate screening adds complexity to the enzyme development process, particularly in terms of screening burden (screening in succession) or analytical requirements (one pot screening), as the analytical method must reliably distinguish between the substrates and formed products.^63^ Since DNA-tagged compounds are expensive and labor-intensive to prepare, enzyme promiscuity could first be evaluated using untagged small molecules, before applying the biocatalysts to on-DNA reactions. For example, the enzymatic cascade for amide bond formation, consisting of CoA-ligases and engineered *N*-acyltransferases, was prefiltered in this way. Engineered variants were first tested on a “decoy” substrate, namely an amine which was attached to a linker moiety but not the DNA. Only variants exhibiting improved activity were further assayed on molecules attached to the DNA tag and, encouragingly, screening results highlighted a good correlation between enzymatic off- and on-DNA activity.^40^ Despite this positive outcome, however, it remains important to consider that the presence of a DNA tag and its linker might interfere with protein–substrate binding.^64–66^ In addition, it is reasonable to expect that DNA architecture may also affect the activity and selectivity of enzymes used in on-DNA synthesis. Indeed, this has been demonstrated in previous studies evaluating different linker lengths, DNA strand designs and the position of the DNA linkage on the small molecule.^38–40^

Selectivity is a further key consideration when choosing a synthetic strategy for DEL construction, as the molecules incorporated during library synthesis often contain multiple functionalities, including those that are contained in the DNA tag itself. Any reaction applied to such complex compounds must be able to selectively target one functional group without affecting others to avoid the formation of undesired side-products, which would compromise the decoding during the selection process. This includes chemoselectivity (choosing between different types of functional groups), regioselectivity (targeting specific positions within the molecule), and stereoselectivity (favoring one stereoisomer over others). DNA-encoded libraries with a regio- and stereodefined presentation of functional groups have proven especially effective for identifying strong binders.^67,68^ In particular, stereoisomers can exhibit significant differences in binding affinity to target molecules.^69^ For example, separately synthesizing and analyzing the two stereoisomers of a BET protein binder, discovered from a DEL containing over 4.5 billion compounds, showed in off-DNA binding assays that the *R*-enantiomer was 50 times more potent than the *S*-enantiomer.^70^ Building on this knowledge, enantiopure starting materials were used to construct a stereodefined DEL. Screening of this library led to the isolation of potent ligands against protein homologues with 10- to 1,000-fold differences in binding affinity between stereoisomers, confirming the contribution of stereodefined elements to library productivity.^69^ These examples highlight the profound impact of chirality on molecular recognition and the importance of implementing selective reactions in DEL synthesis, which are currently lacking.

In this vein, many of the chemical reactions which are currently applied to build complexity on DNA are not chemo- and regioselective, rendering the use of protection group chemistry necessary.^71–73^ Intrinsically, this requirement introduces additional reaction steps, thereby increasing the risk of side-product formation and DNA damage. For instance, while Boc protection is widely used in synthetic chemistry and would be highly attractive for DEL synthesis due to the large number of commercially available Boc-protected building blocks,^36^ the acidic conditions required for efficient Boc-deprotection are mostly incompatible with DNA.^71,74^ As a case in point, micellar Brønsted acid-promoted Boc-deprotection of DNA-conjugated molecules led to DNA depurination of varying extents, preventing reliable decoding of the library.^74^ Alternative deprotection strategy, such as thermal treatment (90 °C), was tested for Boc-phenylalanine DNA-conjugates but reduced the efficiency of subsequent encoding ligations by ∼ 30%.^75^ Although alternative protecting group strategies are available (e.g., Fmoc), the employment of selective reactions, which eliminate the need for protection steps altogether, would be preferable.

Enzymes are known for their remarkable chemo-, regio-, and stereoselectivity. The high selectivity arises from the enzyme’s three-dimensional structure, which controls both the accessibility to and orientation of substrates within the active site.^76,77^ This precision has been favorably played out in the available studies on enzymatic transformation with DNA-conjugated substrates (*vide supra*). For instance, galactose oxidase (GOase) selectively oxidized the primary alcohol (Scheme 1) on the sugar moiety, leaving the DNA headpiece untouched.^37^ Likewise, threonine aldolases showed excellent control over stereochemistry at the α-carbon of the produced β-hydroxy-α-amino acids (Scheme 2).^38^

## Conclusions

DEL syntheses combining biocatalytic and chemical steps^37–40^ have already begun to enlarge the accessible chemical space on DNA illustrating the powerful synergy between biocatalysis and synthetic chemistry,^78^ echoing the well-documented success of enzyme integration in the production of active pharmaceutical ingredients.^79–82^ Considering this, we predict that enzymes will become useful tools for the construction of DNA-encoded chemical libraries in the coming years, improving DEL quality and expanding the accessible chemical space on DNA. Particularly, reactions for which conventional synthetic methods fall short in terms of yield, selectivity, or DNA-compatibility will benefit from biocatalysis.

DEL syntheses combining biocatalytic and chemical steps have already begun to enlarge the accessible chemical space on DNA illustrating the powerful synergy between biocatalysis and synthetic chemistry, echoing the well-documented success of enzyme integration in the production of active pharmaceutical ingredients.

Although concerns about limited substrate scope might remain, recent advances in protein engineering have shown that enzyme promiscuity can be broadened. Looking ahead, advances in bioinformatics will dramatically accelerate enzyme design and evolution, making biocatalysis more accessible and efficient, particularly for complex applications. Enzyme structures can be predicted and visualized with tools such as AlphaFold2 ^83^ and RoseTTAFold,^84^ even in the absence of crystal structures. Further emerging applications are able to predict enzyme–substrate complexes directly from amino acid sequences and SMILES strings,^85,86^ offering new opportunities to understand active site geometry and calculate pocket size, guiding enzyme selection for DEL synthesis. Furthermore, predicting protein properties such as thermostability and solubility narrows down the mutational search space, in this way reducing the need for exhaustive screening.^87^ When combined with high-throughput screening, this strategy accelerates the development of efficient enzymes.^88^

By integrating state-of-the-art experimental and in silico tools for enzyme discovery and engineering into DEL workflows, the enzyme-powered synthesis of more selective and diverse DNA-encoded libraries will soon become a reality.

In summary, harnessing biocatalysis for DEL synthesis is no longer a distant promise but an emerging reality. By integrating state-of-the-art experimental and *in silico* tools for enzyme discovery and engineering^58,89^ into DEL workflows, the enzyme-powered synthesis of more selective and diverse DNA-encoded libraries will soon become a reality.

## Supplementary Material



## Notes

Transparent Peer Review report available (PDF)

## ⊥

Peter Cremer Holding GmbH & Co. KG Glockengiesserwall 3, 20095 Hamburg

## ∇

Chemical and Analytical Development, Novartis Pharma AG, 4056-CH Basel, Switzerland.

## Abbreviations

**DNA**: Deoxyribonucleic acid
**DEL**: DNA-encoded library
**HTS**: High-throughput screening
**PCR**: Polymerase chain reaction
**sEH**: Soluble epoxide hydrolase
**HP**: Headpiece
**β1,4-GalT**: Bovine β1,4-galactosyltransferase
**UDP-Gal**: Uridine diphosphate galactose
**GOase**: Galactose oxidase
**TAs**: Threonine aldolases
***E. coli***: *Escherichia coli*
**OPA**: *ortho*-Phthaldialdehyde
**PEG**: Polyethylene glycol
**PNPCl**: *p*-Nitrophenylchloroformate
**DMTMM**: 4-(4,6-dimethoxy-1,3,5-triazin-2-yl)-4-methyl-morpholinium chloride
**LCMS**: Liquid chromatography coupled to mass spectrometry
**PHGDH**: Phosphoglycerate dehydrogenase
**CL**: CoA ligase
**NAT**: *N*-acyltransferase
**TDC**: Tryptophan decarboxylase
**BET**: Bromodomain and Extra-Terminal motif
