# Supplementary material for: Toward the Chemoenzymatic Synthesis of DNA-Encoded Libraries
Source: ACS Cent Sci. 2026 Jan 6;12(1):28–39. doi: 10.1021/acscentsci.5c01516 (PMC12871881; doi:10.1021/acscentsci.5c01516)
Supplement: Supplementary file 1 [file oc5c01516_si_001.pdf]

oc-2025-015169.R1

Name: Peer Review Information for "Towards the Chemoenzymatic Synthesis of DNA-encoded Libraries"

First Round of Reviewer Comments

Reviewer: 1

Comments to the Author

This perspective discusses how DNA-encoded libraries (DELs)—collections of small molecules tagged with DNA barcodes—have become a central tool in drug discovery by enabling rapid, large-scale ligand screening. However, the chemical reactions routinely used to build DELs are restricted by the need for orthogonal chemistry tolerating DNA tags, which limits library diversity. The authors highlight biocatalysis as a promising way to overcome these challenges. Enzymes operate under mild, DNA-compatible conditions and offer high chemo-, regio-, and stereoselectivity, potentially enabling the synthesis of more complex, diverse, and high-quality DELs. The paper reviews early examples of enzymatic DEL synthesis, outlines key challenges such as substrate scope and DNA compatibility, and points to opportunities in protein engineering, directed evolution, and computational tools to expand enzyme utility. The authors predict that combining enzymatic and chemical methods will soon make biocatalysis a practical and powerful component of DEL construction, broadening accessible chemical space and improving hit discovery in drug development. Overall, this is a novel topic that should be highlighted and is likely to be of interest to the growing DEL community.

Minor issues: please check sugar stereochemistry in Scheme 1 - all monosaccharides appear to be shown as the wrong (non-natural) enantiomers

Reviewer: 2

Comments to the Author

I was quite excited to read a manuscript entitled 'Enzymatic synthesis of DNA-encoded libraries', however, felt quite disappointed after reading the manuscript.

Adding biocatalysis to the synthetic toolbox for the generation of DNA-encoded libraries (DELs) is clearly a very important topic with a potentially exciting perspective. But the title already makes promises that cannot be met and the manuscript lacks depth. Only two literature examples (Scheme 1) from 2017 & 2021 have been presented, which are in fact chemo-enzymatic syntheses — not (fully) enzymatic as stated in the title.

Of course, the authors could compensated for this by providing ideas and potential strategies to change that or add to the current state-of-the-art, in order to advance the field and fuel the imagination of scientists.

After all, the author guidelines of ACS Central Science state that Outlooks should be "visionary, forward-looking, accessible and of general interest".

However, this has been inadequately fulfilled.

The rest of the manuscript only comprises of key features for enzymes to be aware of, look out for, or even laboriously obtain via enzyme engineering for potential future applications in DELs. A large section elaborates on the potential to engineer enzymes to accept many different substrates. A few interesting studies on enzymatic substrate promiscuity are named, but it is not immediately obvious why this is needed: in their two presented literature examples (Scheme 1), biocatalysis is only used for the attachment of the oligo to the building block. Consequently, the enzymes use in this first step only the same substrate. It is also of note, that many of these named studies use enzymes for bond-breaking reactions (hydrolytic P–C bond cleavage, decarboxylation), not bond-forming ones (decarboxylative aldolase), although molecular assembly is needed for DELs as also nicely illustrated in Figure 1 by the authors themselves.

The manuscript closes with biocatalytic transformations of pharmaceuticals that have outperformed chemical equivalents (albeit only slightly), which appears abstract in context

of DELs. Is this manuscript advocating biocatalysis as a complementary tool (Scheme 1) or as a replacement of chemical synthesis (Scheme 2)? In the context of 85% yield, are these 7-10% yield improvement in the Montelukast example (Scheme 2b) really the reason why enzymes should be used in DELs?

In the final outlook, the authors predict that "enzymes will become useful tools for the construction of DNA-encoded chemical libraries in the coming years", but are short of evidence, considering the two literature examples are from 2017 & 2021 and no visionary strategies have been provided.

They further claim that "A hybrid strategy for DEL construction, which combines biocatalytic and chemical steps, can further enlarge the accessible chemical space on DNA.", although all the authors have been showing is exactly such hybrid strategy with their two literature examples (Scheme 1).

Therefore, an in-depth red line is missing and the manuscript does not (yet) provide a visionary perspective for a general interest to the broad readership of ACS Central Science. It is indicative that the authors chose to illustrate enzyme engineering (Figure 2) and a comparison between biocatalytic vs chemical efficiency (Scheme 2) in two of their four figures, rather than truly innovative ideas for the "Enzymatic synthesis of DNA-encoded libraries".

Nevertheless, the topic is highly relevant and the authors should be encouraged to improve their manuscript. Careful revision is recommended keeping in mind central questions such as:

Why are there only two literature examples from more than 4 years ago? What are major drawbacks of the integration of biocatalysis?

Could enzymes provide diversity, rather than chemical methods or are they only useful for oligo attachment?

How could engineered or naturally promiscuous enzymes be integrated into the generation of DELs as advertised? Please give examples.

Below are further, more detailed comments:

Abstract

- The ScholarOne Manuscript submission table says the manuscript type is an "Outlook", while in the abstract the authors have written "In this perspective".

- Must the chemical reaction really need to proceed with high selectivity to minimise side product formation? Is it not the point of DEL synthesis to generate high diversity? Hence, can it not be advantageous to produce several (side-)products?

page 1 line 47

reference 1 is the Health at a Glance report about key indicators for population health and health system performance across OECD member countries — is this really the right reference for the R&D investment of the pharmaceutical industry?

page 1 line 57 "screening as many compounds as possible is a typical first step"

Not sure this would be the first step "in the drug development process". This statement may also mislead the reader: In the past two decades, the focus of the industry has increasingly shifted from ultra-HTS of synthetic compounds to small(er) focused libraries designed with diversity-oriented syntheses (DOS) / more "natural product-like". This is due to reasons such as the "substantial material requirements" listed by the authors later. See for example the reviews by D. J. Newman, G. M. Cragg in J. Nat. Prod. in 2016, 79, 629–661 & 2020, 83, 770–803

Reference 2 for HTS is also from 1996 as a potential reason for this misconception. It sure does not show whether the authors know "current HTS techniques" as stated in the next line.

page 3 lines 33

"DEL synthesis to 2-4 cycles" — here numerically, in the caption of Figure 1 you spell out the numbers

page 3 lines 12-26

The description for the generation of the diversity of both the compound library and the DNA-barcode could be improved for the reader's understanding:

- Does the first set of chemical building blocks react with the oligo or its functional handle?

- How large is such a set i.e. how many of the same oligo-building blocks conjugates are present?

- How many specific DNA fragments are there? One for each oligo? Probably not if you indicate further DNA ligation taking place. However, in Figure 1 you indicate that the following ligation steps occur all with the same DNA fragment (yellow-red DNA). Or do these indicate the sub-libraries for hybridisation?

- Are these always unique DNA-tags or can there be two compounds having the same tags, which can be disregarded because of the following filtering steps of binder / non-binder molecules?

- The term "chemical synthesis" indicates a route to a certain product — erhaps derivatisation / diversification is a better description.

- "Billions of compounds" — different / individual

page 3 line 53

While the library generation has been comprehensively described, a description of downstream processes of the library is missing. The second half of Figure 1 (starting with the "panning" step) has only been described in the caption, not in the text. However, this is of course a key step, considering the effort put into library generation — for what reason?

page 4 line 10

Please give reasons why the listed conditions or reagents should be avoided in the presence of DNA. What damage / side-reaction could occur?

At page 4 line 23

It is true that many on-DNA reactions will result in damage, which is an argument for more benign techniques such as biocatalysis. However, the authors list Pd-catalysis as

especially harmful with only 0.1% of DNA remaining. Indeed, the cited article states this very case. However, by simply changing the order of reagent addition, this was improved to 40%. Also in the provided tables, none of the Pd-catalysed reactions listed run so badly. So this feels a little like cherry-picking. I believe there is consensus among chemists that 0.1% amplifyable DNA is not an acceptable level of damage for DELs.

page 4 line 44

"biocatalysis could be" — could be or is?

page 4 line 46

"enzymes are powerful catalyst" — catalysts

page 5 line 24

Figure 1 — the DNA tag is shown as double-stranded DNA. Is that really the case for DELs? No comment has been made in the text about this.

page 5 chapter "Biocatalysis for the construction of DELs"

I am missing the term "chemo-enzymatic" in this chapter. It should be made clear to the reader that biocatalysis is only the tool for conjugation to the DNA code, while chemical reactions provide diversification. In fact, in Scheme 1a, compound 1 was only extended. How was compound 1 generated? Could the authors also provide an explanation why enzymes were not use for the diversification reactions?

page 10 line 12 "To date, enzymatic transformations using DNA-conjugated substrates have not been associated with DNA degradation"

This statement is not particularly impressive, considering only three studies from 2016, 2017 and 2021 are cited (and known).

page 10 line 34 "the applied enzymes must be able to catalyze reactions between hundreds of different molecules"

This is an ambiguous statement. Do the enzymes catalyse reactions between all these molecules? Or do the enzymes just need to accept different substrates? Why do they need to be able to do this? In your examples in Scheme 1, they all use the same substrate…

page 11 line 55

How are tryptophan decarboxylase going to help assemble diverse DELs?

page 12 line 12

Please show how decarboxylative aldolases could assemble diverse DELs rather than explaining directed evolution.

page 12 line 40–48

"Since DNA-tagged compounds are expensive and labor-intensive to prepare, enzyme promiscuity could first be evaluated using untagged small molecules" — how likely are enzymes to accept substrates with a linker/oligo attached to the substrate, which is bound in tight enzyme pockets? Please comment on that to give an idea if a directed evolution campaign is worth pursuing on the substrate without, and later with the oligo.

page 12 line 5–13

Is is not important to distiguish between the first DEL and the second DEL? In the first, lax stereoselectivity may be the key to produce diversity i.e. both stereoisomers (or install the functional group at all), in order to get a hit. Of course, selectivity becomes important if only one isomer is desired.

page 14 line 37

An Fmoc protecting group could be used instead of Boc to protect amines.

Scheme 2

I am not sure how this fits into the topic of DELs. Why the comparison? Please show an example of how this is important for DNA-encoded libraries.

Outlook

Please see above.

Author's Response to Peer Review Comments:

Reviewer #1 (Remark to Authors)

This perspective discusses how DNA-encoded libraries (DELs)—collections of small molecules tagged with DNA barcodes—have become a central tool in drug discovery by enabling rapid, large-scale ligand screening. However, the chemical reactions routinely used to build DELs are restricted by the need for orthogonal chemistry tolerating DNA tags, which limits library diversity. The authors highlight biocatalysis as a promising way to overcome these challenges. Enzymes operate under mild, DNAcompatible conditions and offer high chemo-, regio-, and stereoselectivity, potentially enabling the synthesis of more complex, diverse, and high-quality DELs. The paper reviews early examples of enzymatic DEL synthesis, outlines key challenges such as substrate scope and DNA compatibility, and points to opportunities in protein engineering, directed evolution, and computational tools to expand enzyme utility. The authors predict that combining enzymatic and chemical methods will soon make biocatalysis a practical and powerful component of DEL construction, broadening accessible chemical space and improving hit discovery in drug development. Overall, this is a novel topic that should be highlighted and is likely to be of interest to the growing DEL community.

We are grateful to Reviewer 1 for reviewing our manuscript and their valuable feedback on how to improve it.

1) Please check sugar stereochemistry in Scheme 1 - all monosaccharides appear to be shown as the wrong (non-natural) enantiomers

We thank Reviewer #1 for catching this error. Scheme 1 has been corrected to display the correct enantiomers of the monosaccharides.

Reviewer #2 (Remark to Authors)

We thank Reviewer 2 for the detailed and constructive feedback. We carefully considered each comment and substantially revised the manuscript to clarify our message, improve coherence, and strengthen the forward-looking aspects of the Outlook on how biocatalysis can enhance DEL synthesis.

1) I was quite excited to read a manuscript entitled 'Enzymatic synthesis of DNA-encoded libraries', however, felt quite disappointed after reading the manuscript. Adding biocatalysis to the synthetic toolbox for the generation of DNA-encoded libraries (DELs) is clearly a very important topic with a potentially exciting perspective. But the title already makes promises that cannot be met and the manuscript lacks depth.

We agree that the original title overstated the maturity of the field. To better reflect the exploratory nature of our Outlook, we have revised the title to:

“Towards Chemoenzymatic Synthesis of DNA-Encoded Libraries.”

2) Only two literature examples (Scheme 1) from 2017 & 2021 have been presented, which are in fact chemo-enzymatic syntheses — not (fully) enzymatic as stated in the title.

At the time of initial submission, the two examples we discussed were the only published studies that apply enzymatic transformations to DEL synthesis. However, we can now add two more studies which appeared as preprints in the meantime (Buller/Scheuermann labs: https://doi.org/10.21203/rs.3.rs7598475/v1 and Xiaojie Lu lab: 10.26434/chemrxiv-2025-nln78) highlighting the rapid progress that this field is making, including the use of engineered enzymes for C-N bond formation. We discuss both studies in the main text and added additional figure elements.

3) Of course, the authors could compensate for this by providing ideas and potential strategies to change that or add to the current state-of-the-art, in order to advance the field and fuel the imagination of scientists. After all, the author guidelines of ACS Central Science state that Outlooks should be "visionary, forward-looking, accessible and of general interest".
However, this has been inadequately fulfilled.

The rest of the manuscript only comprises of key features for of, look out for, or even laboriously obtain via enzyme engineering for potential future applications in DELs. A large section elaborates on the potential to engineer enzymes to accept many different substrates. A few interesting studies on enzymatic substrate promiscuity are named, but it is not immediately obvious why this is needed: in their two presented literature examples (Scheme 1), biocatalysis is only used for the attachment of the oligo to the building block. Consequently, the enzymes use in this first step only the same substrate.

We are sorry to hear that the forward-looking aspect of the perspective was not as clearly discernible as we had hoped. To strengthen the forward-looking aspect of our perspective further, we expanded the discussion to include strategies for integrating and engineering enzymes for DEL chemistry. In addition, we added further examples of C–C and C–N bond-forming enzymes (e.g., aldolases and amide bond synthetases) that could be adapted for DNA-compatible synthesis. These examples illustrate how biocatalysis can complement and extend the existing DEL reaction portfolio. Importantly, we would like to stress that enzymes are used well-beyond attaching the oligo to the first building block but can be harnessed to create diversity in any of the subsequent DEL cycles.

We also restructured the discussion to explicitly connect substrate promiscuity and enzyme engineering to the requirements of DEL construction, clarifying why these features are critical for enabling enzymatic diversity generation.

We have split the previous Scheme 1 into two schemes:

- Scheme 1: Chemoenzymatic construction of a carbohydrate-derived DEL
- Scheme 2: Enzymatic formation of β-hydroxy amino acid-derived DELs with further chemical diversification

Each scheme now includes a workflow overview illustrating which steps were enzymatic and how they contributed to library diversification rather than mere DNA attachment. These revisions clarify why enzyme promiscuity is essential and how biocatalysis was used to introduce new building blocks.

4) It is also of note, that many of these named studies use enzymes for bond-breaking reactions (hydrolytic P–C bond cleavage, decarboxylation), not bond-forming ones (decarboxylative aldolase), although molecular assembly is needed for DELs as also nicely illustrated in Figure 1 by the authors themselves.

Our intention in including these examples was to illustrate the principles and success of enzyme engineering for substrate scope expansion, rather than to suggest their direct use in DEL synthesis. We have clarified this in the text and supplemented the section with an additional example of an amide bond synthetase (ABS), published in 2025 (doi: 10.1038/s41467-024-55399-0), which is directly relevant to DEL-style bond-forming chemistry.

5) The manuscript closes with biocatalytic transformations of pharmaceuticals that have outperformed chemical equivalents (albeit only slightly), which appears abstract in context of DELs. Is this manuscript advocating biocatalysis as a complementary tool (Scheme 1) or as a replacement of chemical synthesis (Scheme 2)? In the context of 85% yield, are these 7-10% yield improvement in the Montelukast example (Scheme 2b) really the reason why enzymes should be used in DELs?

Thank you for this comment. We have removed these examples, including the Figure, and adapted the text to clarify that our perspective presents biocatalysis as a complementary tool in DEL synthesis, not as a replacement for chemocatalysis

6) Therefore, an in-depth red line is missing and the manuscript does not (yet) provide a visionary perspective for a general interest to the broad readership of ACS Central Science. It is indicative that the authors chose to illustrate enzyme engineering (Figure 2) and a comparison between biocatalytic vs chemical efficiency (Scheme 2) in two of their four figures, rather than truly innovative ideas for the "Enzymatic synthesis of DNA-encoded libraries".

We revised the manuscript to provide a clearer narrative:

1. **Introduction** to DEL chemistry and its limitations.
2. **Enzymatic Strategies.** Review of the current state of applying biocatalysis in DEL synthesis (including a description of the now available two preprints)
3. **Challenges and Opportunities:** lessons from existing studies, substrate promiscuity, DNA compatibility, selectivity.
4. **Conclusion**

We believe the revised structure now better conveys a coherent, forward-looking message.

7) Nevertheless, the topic is highly relevant and the authors should be encouraged to improve their manuscript. Careful revision is recommended keeping in mind central questions such as: Why are there only two literature examples from more than 4 years ago? What are major drawbacks of the integration of biocatalysis?

As delineated in our answer to point 2, we now have included two additional manuscripts which appeared as preprints in the few months since initial submission of our Outlook highlighting the accelerating interest of using enzymes in DEL construction. In addition, we explicitly discuss the major limitations of integrating biocatalysis into DEL synthesis, such as bulky DNA-tag acceptance, limited substrate scope, and suggest potential approaches to overcome these, including the use of enzymes with large active sites or engineering for increased flexibility and promiscuity.

8) Could enzymes provide diversity, rather than chemical methods or are they only useful for oligo attachment? How could engineered or naturally promiscuous enzymes be integrated into the generation of DELs as advertised? Please give examples.

All available literature examples use enzymes to generate diversity beyond simple conjugation to the DNA tag. We have expanded the corresponding discussion in the main text to make this aspect clearer for the reader. In addition, we now include a box with the “Strategy” at the top of scheme 1, 2 and 3 to highlight at which point in DEL construction the enzymatic step is employed.

- In the carbohydrate DEL, enzymatic oxidation enabled site-specific modification of highly functionalized substrates, enabling subsequent diversification not easily achieved chemically.
- In the β-hydroxy amino acid DEL, enzymatic C–C coupling generated structurally diverse DNA conjugates containing multiple functional handles for further library elaboration.
- In the study with a decarboxylative aldolase, the enzymatic step was employed for scaffold preparation by C-C bond formation, enabling subsequent building block coupling.
- In the CoA Ligase/NAT DEL, enzymatic amide bond formation allowed the conjugation of diverse carboxylic acid building blocks both in the first and in the second cycle of split-and-pool synthesis. The enzymes accepted very bulky and diverse substrates yielding coupling products with excellent yields (on average > 90 %).

Below are further, more detailed comments:

9) The ScholarOne Manuscript submission table says the manuscript type is an "Outlook", while in the abstract the authors have written "In this perspective".

We have corrected the manuscript type in the text from “Perspective” to “Outlook.”

10) Must the chemical reaction really need to proceed with high selectivity to minimise side product formation? Is it not the point of DEL synthesis to generate high diversity? Hence, can it not be advantageous to produce several (side-)products?

While diversity is central to DEL synthesis, uncontrolled side reactions create mixtures of compounds sharing the same DNA barcode, thereby compromising decoding accuracy. Hence, reactions must be selective toward the intended product while still being broadly applicable across different substrates. We clarified this distinction in the revised text. Please also see below for a graphical representation of the problem of side reactions.

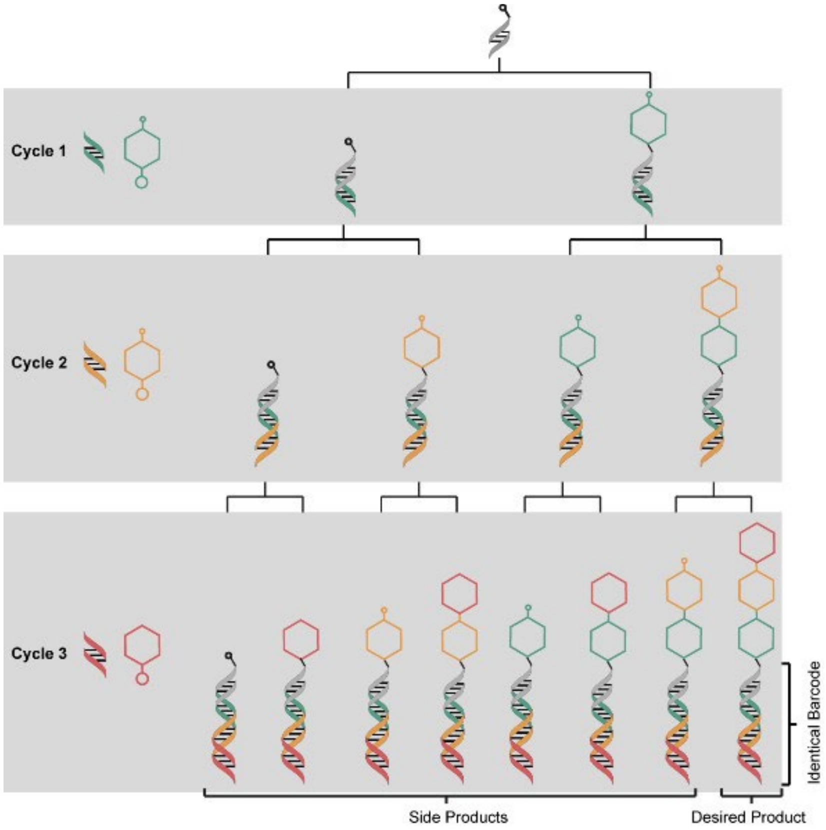

11) page 1 line 47: reference 1 is the Health at a Glance report about key indicators for population health and health system performance across OECD member countries — is this really the right reference for the R&D investment of the pharmaceutical industry?

The OECD report dedicates a whole section on pharmaceutical research and development (starting from page 206) and gives numbers for the R&D expenditures of the pharmaceutical industry for research and development. While the report is quite comprehensive already, we added an additional reference (Wouters, O. J.; McKee, M.; Luyten, J. Estimated Research and Development Investment Needed to Bring a New Medicine to Market, 2009-2018. JAMA 2020, 323 (9), 844. https://doi.org/10.1001/jama.2020.1166.) to further underline the cost of drug development.

12) page 1 line 57 "screening as many compounds as possible is a typical first step" Not sure this would be the first step "in the drug development process". This statement may also mislead the reader: In the past two decades, the focus of the industry has increasingly shifted from ultra-HTS of synthetic compounds to small(er) focused libraries designed with diversity-oriented syntheses (DOS) / more "natural product-like". This is due to reasons such as the "substantial material requirements" listed by the authors later. See for example the reviews by D. J. Newman, G. M. Cragg in
J. Nat. Prod. in 2016, 79, 629–661 & 2020, 83, 770–803

Reference 2 for HTS is also from 1996 as a potential reason for this misconception. It sure does not show whether the authors know "current HTS techniques" as stated in the next line.

We have updated the HTS section with recent references and revised wording to reflect the current focus on smaller, diversity-oriented libraries.

13) page 3 lines 33

"DEL synthesis to 2-4 cycles" — here numerically, in the caption of Figure 1 you spell out the numbers

We have adapted the spelling in the main text.

14) page 3 lines 12-26

The description for the generation of the diversity of both the compound library and the DNA-barcode could be improved for the reader's understanding:

- Does the first set of chemical building blocks react with the oligo or its functional handle?

The building blocks reacts with the functional handle of the oligo. We now specify this in the text.

- How large is such a set i.e. how many of the same oligo-building blocks conjugates are present?

We have added a quantitative example to the main text illustrating how combinatorial cycles yield multimillion-member libraries. In each case, the synthesis is conducted at a very small scale as only picomolar library quantities are needed for the selection process. Recovery and identification of binders is possible with 10^5 copies of an individual compound.

- How many specific DNA fragments are there? One for each oligo? Probably not if you indicate further DNA ligation taking place. However, in Figure 1 you indicate that the following ligation steps occur all with the same DNA fragment (yellow-red DNA). Or do these indicate the sub-libraries for hybridisation? Are these always unique DNA-tags or can there be two compounds having the same tags, which can be disregarded because of the following filtering steps of binder / non-binder molecules?

We have revised Figure 1 to make the DEL construction process more intuitive. In essence, identical building blocks are coupled to the same oligonucleotide. However, distinct final compounds (resulting from multiple synthetic steps involving several building blocks) must not share the same overall DNA tag. Instead, each compound should carry a unique combination of DNA codes that reflect the specific building blocks incorporated into the molecule. To enable reliable identification after panning, the DNA tag must therefore be unique for each compound.

- The term "chemical synthesis" indicates a route to a certain product — perhaps derivatisation / diversification is a better description.

We have adapted the main text to clarify that library synthesis proceeds via building-block coupling, not general “chemical synthesis.”

- "Billions of compounds" — different / individual

We added the word “individual”.

15) page 3 line 53

While the library generation has been comprehensively described, a description of downstream processes of the library is missing. The second half of Figure 1 (starting with the "panning" step) has only been described in the caption, not in the text. However, this is of course a key step, considering the effort put into library generation — for what reason?

Thank you for this suggestion. We have revised the description of Figure 1 to include an explanation of downstream selection (panning) steps.

16) page 4 line 10

Please give reasons why the listed conditions or reagents should be avoided in the presence of DNA. What damage / side-reaction could occur?

We have added a more detailed discussion of DNA damage types (cleavage, depurination, base transversion) and how these relate to reaction conditions.

17) At page 4 line 23

It is true that many on-DNA reactions will result in damage, which is an argument for more benign techniques such as biocatalysis. However, the authors list Pd-catalysis as especially harmful with only 0.1% of DNA remaining. Indeed, the cited article states this very case. However, by simply changing the order of reagent addition, this was improved to 40%. Also in the provided tables, none of the Pdcatalysed reactions listed run so badly. So this feels a little like cherry-picking. I believe there is consensus among chemists that 0.1% amplifyable DNA is not an acceptable level of damage for DELs.

We apologize for this oversight. For the Pd-catalysis example we have now specified that modifying the reagent order significantly enhances DNA recovery.

18) page 4 line 44

"biocatalysis could be" — could be or is?

Given the currently limited number of studies exploring biocatalysis in the context of DNA-encoded library synthesis, we intentionally used the more cautious phrasing “could be.” While we are confident that biocatalysis holds strong potential as a synthetic approach for DEL construction,

further experimental validation is required before making a definitive claim. The purpose of this Outlook is to draw attention to this promising area and to stimulate further research.

19) page 4 line 46: "enzymes are powerful catalyst" — catalysts

We corrected this typo.

20) page 5 line 24: Figure 1 — the DNA tag is shown as double-stranded DNA. Is that really the case for DELs? No comment has been made in the text about this.

DNA encoded libraries can be constructed using either double or single stranded DNA tags. To clarify which type of DNA tag was used in each of the discussed studies, we added the structure of the applied DNA-headpieces/tag to the respective schemes.

21) page 5 chapter "Biocatalysis for the construction of DELs" I am missing the term "chemo-enzymatic" in this chapter. It should be made clear to the reader that biocatalysis is only the tool for conjugation to the DNA code, while chemical reactions provide diversification. In fact, in Scheme 1a, compound 1 was only extended. How was compound 1 generated? Could the authors also provide an explanation why enzymes were not use for the diversification reactions?

Please let us clarify this misconception: Enzymes were **not** used for the conjugation of the initial scaffold to the DNA tag; this step was performed chemically. In the carbohydrate library example, the enzymes were applied to extend a chemically pre-installed sugar moiety by adding different sugar building blocks (yielding seven distinct DNA-conjugated molecules) and to catalyze site-specific oxidations that enabled further chemical diversification. While this was described in the main text, we agree that the previous version of our figures did not clearly illustrate the chemoenzymatic workflow. We have therefore revised Schemes 1 - 3 to better depict the chemoenzymatic nature of the library construction, in each case highlighting at which step of library construction the enzymes are employed.

22) page 10 line 12 "To date, enzymatic transformations using DNA-conjugated substrates have not been associated with DNA degradation"

This statement is not particularly impressive, considering only three studies from 2016, 2017 and 2021 are cited (and known).

We agree that currently only four published studies are available to draw conclusions from. However, we would like to point out that in the available reports five distinct enzyme families (threonine aldolases, glycosyltransferases, galactose oxidase, N-acyltransferases and CoA ligases) were used on a variety of different substrates all leading to the same conclusion, namely that enzymes are particularly mild to DNA-conjugated substrates.

Nevertheless, in the revised text, we clarify that while enzymes often operate under conditions generally considered DNA-compatible (neutral pH, ambient temperature, aqueous media), the compatibility of each biocatalyst must still be evaluated on a case-by-case basis. We also emphasize that not all enzymes will necessarily tolerate DNA-linked substrates.

23) page 10 line 34 "the applied enzymes must be able to catalyze reactions between hundreds of different molecules"

This is an ambiguous statement. Do the enzymes catalyse reactions between all these molecules? Or do the enzymes just need to accept different substrates? Why do they need to be able to do this? In your examples in Scheme 1, they all use the same substrate...

We have revised Scheme 1 to better illustrate that, in both examples (Carbohydrate and b-hydroxy amino acid), the enzymes catalyse reactions with different building blocks rather than a single substrate. Accordingly, we have clarified in the text that enzymes intended for DEL synthesis must exhibit a certain degree of substrate promiscuity, allowing them to accept and transform a range of substrates rather. We further emphasize that such flexibility is essential for real-world DEL applications, where diverse building blocks are required to generate large and chemically varied libraries.

24) page 11 line 55: How are tryptophan decarboxylase going to help assemble diverse DELs?

The purpose of including tryptophan decarboxylase in this section was not to suggest its direct use in

DEL synthesis but rather to illustrate how directed evolution can be employed to expand substrate promiscuity in enzymes. This example serves to demonstrate the general strategy of tailoring enzyme specificity and activity, which could be applied to develop biocatalysts suitable for DEL construction. We added a second example where with a similar approach engineering was conducted with a decarboxylative aldolase, an enzyme class that was also used in DEL synthesis previously.

25) page 12 line 12: Please show how decarboxylative aldolases could assemble diverse DELs rather than explaining directed evolution.

Our primary goal in this section was to illustrate how enzyme engineering can be applied to enhance substrate promiscuity, which we consider one of the main limitations for the broader application of biocatalysts in DEL synthesis. But it’s of course correct that decarboxylative aldolases themselves could be directly valuable for DEL construction as these enzymes enable C–C bond formation between amino acid side chains and ketones or aldehydes, leading to the generation of γ-hydroxy amino acid derivatives. We now mention this important aspect in the main text.

26) page 12 line 40–48

"Since DNA-tagged compounds are expensive and labor-intensive to prepare, enzyme promiscuity could first be evaluated using untagged small molecules" — how likely are enzymes to accept substrates with a linker/oligo attached to the substrate, which is bound in tight enzyme pockets? Please comment on that to give an idea if a directed evolution campaign is worth pursuing on the substrate without, and later with the oligo.

Immediately following the cited sentence, we note that “the presence of a DNA tag and its linker can interfere with protein–substrate binding,” acknowledging the potential challenge of screening with untagged substrates. However, given that DNA-tagged compounds are expensive and time-consuming to prepare, particularly in academic settings, we propose an initial screening of enzyme promiscuity using untagged small molecules as a practical first step. In fact, this approach has succefully been employed in a recent study from our lab (https://doi.org/10.21203/rs.3.rs-7598475/v1) and which we now describe to illustrate this point.

27) page 12 line 5–13

Is is not important to distiguish between the first DEL and the second DEL? In the first, lax stereoselectivity may be the key to produce diversity i.e. both stereoisomers (or install the functional group at all), in order to get a hit. Of course, selectivity becomes important if only one isomer is desired.

While generating diversity is essential in DEL construction, insufficient stereoselectivity produces mixtures of enantiomers that share a single DNA barcode, obscuring hit attribution during selection. In a racemic outcome, only ~50% of the barcoded material corresponds to the active enantiomer (if only one enantiomer binds), effectively diluting its copy number and reducing the likelihood of recovery and accurate ranking. Biocatalysis offers a practical route to install stereochemistry under DNA-compatible conditions, reducing ambiguity at the selection stage.

28) page 14 line 37

An Fmoc protecting group could be used instead of Boc to protect amines.

We now justify the use of Boc protection based on the abundance of commercially available Bocprotected building blocks and indicate that Fmoc use is a possibility.

29)Scheme 2

I am not sure how this fits into the topic of DELs. Why the comparison? Please show an example of how this is important for DNA-encoded libraries.

Following the suggestion of Reviewer 2, Scheme 2 has been removed.

Reponse to Editorial Feedback

1) Author Affiliations: Please include author affiliations to the first page of the manuscript under the author list. Please use separate labels for each affiliation next to the authors' names.

Author affiliations were added at the first page beneath the author list with separate labels for the different affiliations.

2) Author List: Please include the email address(es) of the corresponding author(s) on the first page of the manuscript.

We added the email address of the corresponding author.

3) Pull Quotes (Outlook): We encourage you to select 3 - 4 quotes from your Outlook that you would like highlighted in your paper. The quotes should be one sentence-long, unique to the Outlook and not from previously cited work. Please list your quotes at the end of the manuscript file.

Quote 1: Regardless of the strategy chosen for DEL assembly, the chemistry that can be applied to prepare the library is restricted by the stability of the DNA tag.

Quote 2: Considering these synthetic restrictions, biocatalysis could be a particularly attractive addition to the DEL reaction portfolio, as enzymes are powerful catalysts naturally operating under "DNAfriendly" conditions.

Quote 3: DEL syntheses combining biocatalytic and chemical steps have already begun to enlarge the accessible chemical space on DNA illustrating the powerful synergy between biocatalysis and synthetic chemistry, echoing the well-documented success of enzyme integration in the production of active pharmaceutical ingredients.

Quote 4: By integrating state-of-the-art experimental and in silico tools for enzyme discovery and engineering into DEL workflows, the enzyme-powered synthesis of more selective and diverse DNAencoded libraries will soon become a reality.

4) Synopsis: ACS Central Science requires a brief synopsis. The synopsis should be no more than 200 characters (including spaces) and should reasonably correlate with the Table of Contents (TOC) graphic. The synopsis is intended to explain the importance of the article to a broader readership across the sciences. Please place your synopsis in the manuscript file after the TOC graphic and label as "Synopsis."

We added a synopsis after the TOC graphic and labelled it as "Synopsis"

This outlook emphasizes the potential of biocatalysis to broaden the reaction scope of DNA-encoded library synthesis beyond chemocatalysis.
